# Connectomic insights into the impact of 1p/19q co-deletion in dominant hemisphere insular glioma patients

**DOI:** 10.3389/fnins.2024.1283518

**Published:** 2024-07-29

**Authors:** Zuo-cheng Yang, Bo-wen Xue, Xin-yu Song, Chuan-dong Yin, Fang-cheng Yeh, Gen Li, Zheng-hai Deng, Sheng-jun Sun, Zong-gang Hou, Jian Xie

**Affiliations:** ^1^Department of Neurosurgery, Beijing Tiantan Hospital, Capital Medical University, Beijing, China; ^2^Department of Neurological Surgery, University of Pittsburgh, Pittsburgh, PA, United States; ^3^Department of Bioengineering, University of Pittsburgh, Pittsburgh, PA, United States; ^4^Neuroimaging Center, Beijing Tiantan Hospital, Capital Medical University, Beijing, China

**Keywords:** insular gliomas, 1p/19q co-deletion, structural connectivity, graph theoretical networks, tractography

## Abstract

**Objectives:**

This study aimed to elucidate the influences of 1p/19q co-deletion on structural connectivity alterations in patients with dominant hemisphere insular diffuse gliomas.

**Methods:**

We incorporated 32 cases of left insular gliomas and 20 healthy controls for this study. Using diffusion MRI, we applied correlational tractography, differential tractography, and graph theoretical analysis to explore the potential connectivity associated with 1p/19q co-deletion.

**Results:**

The study revealed that the quantitative anisotropy (QA) of key deep medial fiber tracts, including the anterior thalamic radiation, superior thalamic radiation, fornix, and cingulum, had significant negative associations with 1p/19q co-deletion (FDR = 4.72 × 10^–5^). These tracts are crucial in maintaining the integrity of brain networks. Differential analysis further supported these findings (FWER-corrected *p* < 0.05). The 1p/19q non-co-deletion group exhibited significantly higher clustering coefficients (FDR-corrected *p* < 0.05) and reduced betweenness centrality (FDR-corrected *p* < 0.05) in regions around the tumor compared to HC group. Graph theoretical analysis indicated that non-co-deletion patients had increased local clustering and decreased betweenness centrality in peritumoral brain regions compared to co-deletion patients and healthy controls (FDR-corrected *p* < 0.05). Additionally, despite not being significant through correction, patients with 1p/19q co-deletion exhibited lower trends in weighted average clustering coefficient, transitivity, small worldness, and global efficiency, while showing higher tendencies in weighted path length compared to patients without the co-deletion.

**Conclusion:**

The findings of this study underline the significant role of 1p/19q co-deletion in altering structural connectivity in insular glioma patients. These alterations in brain networks could have profound implications for the neural functionality in patients with dominant hemisphere insular gliomas.

## Introduction

Diffuse gliomas, prevalent malignant tumors within the central nervous system, are intricately linked to alterations in the brain’s connectome (Osswald et al., 2015; Ostrom et al., 2019; Stoecklein et al., 2020). Their widespread infiltration, particularly in pivotal regions like the insula, highlights the profound impact of gliomas on neural connectivity patterns. As the field of connectomics delves deeper into the brain’s complex network structures, understanding the molecular intricacies of gliomas becomes paramount (Hart et al., 2019; Douw et al., 2023). A standout marker in this context is the co-deletion of 1p/19q, frequently identified in oligodendrogliomas (Griffin et al., 2006; Nicholson and Fine, 2021). This genetic alteration, frequently identified in oligodendrogliomas, is associated with improved prognosis in WHO grade 2–3 gliomas and serves as a distinguishing hallmark from other gliomas like astrocytomas (Jenkins et al., 2006; Antonelli and Poliani, 2022). Furthermore, this co-deletion plays a significant role in molding the brain’s connectivity, with unique invasion patterns and connectivity alterations (Wesseling et al., 2015; Latini et al., 2020). These nuances set the stage for employing advanced imaging techniques to intricately explore the structural connectivity implications of the 1p/19q co-deletion in insular gliomas.

Diffusion MRI (dMRI) has become instrumental in exploring white matter connectivity in connectomics studies. This importance is underscored by research showing how specific molecular changes in gliomas can affect white matter connections in various ways (Latini et al., 2020, 2021). Generalized q-sampling imaging (GQI) serves as an advanced form of dMRI. It excels at identifying the direction of fiber tracts and measuring how water diffuses within them, even when faced with challenges like tumor-induced swelling. Cutting-edge methods such as correlational and differential tractography have also been introduced to improve the study of fiber tracts (Yeh et al., 2016a,^2019^). Correlational tractography (Yeh et al., 2021), measures the degree of connectivity among neighboring voxels within a white matter fiber tract, as defined by diffusion spin density. This method diverges from traditional techniques by accurately tracking only those fiber tract segments that demonstrate substantial correlations with the research variable, thereby enhancing precision in representing the structure and density of white matter fiber tracts. On the other hand, differential tractography represents an upgrade over traditional techniques (Yeh et al., 2019). It pinpoints specific tract segments by comparing their properties on a voxel-by-voxel basis, focusing particularly on changes that cause significant damage. The volume of these tracts can then be used to assess the extent of early neural damage. Another promising avenue is the application of graph theory to analyze the structural brain networks associated with gliomas (Aerts et al., 2018; Dadario et al., 2023). Graph theory allows for the mapping of nodes (key points in the brain) and edges (the connections between them), providing a holistic view of brain connectivity (Bullmore and Bassett, 2011). Properties like the clustering coefficient, betweenness centrality, and small-worldness are calculated to describe both global and local features of these networks. The clustering coefficient reveals how interconnected a node’s neighbors are, while betweenness centrality indicates how often a node acts as a bridge in the shortest paths across the network. Small-worldness characterizes networks that have both high local clustering and short overall path lengths. These measures describe the network’s features on both global and local scales, the thereby providing an encompassing perspective of the network’s structural and functional dynamics associated with glioma pathophysiology (Bullmore and Sporns, 2009; Bullmore and Bassett, 2011). A prior study demonstrated the link between fiber tract disconnection and motor deficits in glioma patients, informing presurgical risk evaluation (Tuncer et al., 2022). Network analysis showed that glioma patients have lower global and local efficiency in the ipsilesional hemisphere than in the contralesional hemisphere (Fekonja et al., 2022). Additionally, a DTI structural graph network study examined variations in graph theory networks based on different IDH1 statuses (Kesler et al., 2017). While these methods have been predominantly used to investigate structural connectivity in gliomas, their application to study the effects of the 1p/19q co-deletion status on brain structural connectivity in gliomas remains an unexplored territory. This uncharted terrain underscores the necessity for further research to deepen our understanding of the link between genetic alterations and their impact on brain connection and function in glioma patients.

In this study, our primary objective was to investigate the differences in brain connectivity among patients with insular gliomas, with a particular emphasis on those harboring and lacking the 1p/19q co-deletion. By leveraging advanced methods, we were able to explore these variations in structural connectivity in detail. Our study demonstrated a notable association between the values of quantitative anisotropy (QA) in specific tracts and the occurrence of 1p/19q co-deletion, as determined through correlation tractography. QA is a sophisticated MRI metric that quantifies the degree of directional organization within white matter tracts, offering critical insights into the microstructural integrity of neural pathways and their alterations in gliomas (Yeh et al., 2013; Celtikci et al., 2018). We used differential tractography to discern the differences in fiber tract segments associated with varying 1p/19q statuses. These two tractography approaches, closely aligned with the concept of “along-tract statistics,” may offer a more precise framework for segment-wise analysis in gliomas (Yeh et al., 2016a,^2019^; Fekonja et al., 2020). Further, when we compared graph theoretical properties among the groups, we discerned remarkable disparities in local and global properties between patients with the 1p/19q co-deletion and those without it. These findings carry profound implications for the management of insular gliomas. They underscore the possibility of developing more individualized management strategies, tailored to the unique genetic and structural brain characteristics of insular glioma patients.

## Materials and methods

### Participants

In this study, we analyzed 93 insular glioma patients from our institution who underwent preoperative dMRI examinations from July 2019 to May 2023. We excluded patients with a midline shift of more than 1 cm (15 patients), those who had not undergone next-generation sequencing (NGS) testing for 1p/19q status (12 patients), and glioblastomas (17 patients) due to their distinct characteristics per the 2016 WHO glioma classification criteria (Louis et al., 2016). Additionally, we excluded right hemisphere insular gliomas (24 patients) due to the extreme imbalance in the ratio of 1p/19q co-deletion to non-co-deletion, which could bias our results. Ultimately, we included thirty-two cases of WHO grade 2–3 left hemisphere insular diffuse gliomas: 13 with 1p/19q co-deletion and 19 without. For comparison, we recruited 20 age- and gender-matched healthy controls (HC). Both the patient and control groups completed the Edinburgh Handedness Questionnaire to determine right-handedness, either before or after the MRI scan.

Two experts manually delineated the tumor boundaries on T1 or T2-weighted images and determined the tumor volume using DSI-Studio software. We observed no significant differences among these groups in terms of age (*p* = 0.076), sex (*p* = 0.787), tumor volume (*p* = 0.203), and grades (*p* = 0.835). However, there was a significant difference in age (*p* = 0.021) between the tumor groups, while sex did not present a significant difference (*p* = 0.837). Detailed demographic information is presented in Table 1. Before being included in the research, all individuals gave their informed consent in writing. The study has received evaluation and approval from the ethics committee of our hospital (KY 2020-146-02).

**TABLE 1:** Demographics.

	Healthy controls (*n* = 20)	1p/19q co-deletion patients (*n* = 13)	1p/19q non-co-deletion patients (*n* = 19)	*p*-value
Age (years)	40.45 ± 10.79	46.62 ± 9.66	38.42 ± 9.18	0.079^†^
**Sex**				
Male	10 (50.00%)	8 (61.54%)	11 (57.89%)	0.787
Female	10 (50.00%)	5 (38.46%)	8 (42.11%)	
Tumor volume (mm3)	NA	44,094.962 ± 29,495.364	62,008.463 ± 43,152.760	0.203
**Pathological grades**				0.835
II grade	NA	10 (76.92%)	14 (73.68%)	
III grade	NA	3 (23.08%)	5 (26.32%)	
IDH status				0.132
Wild type	NA	0 (0.00%)	3 (15.79%)	
Mutation	NA	13 (100.00%)	16 (84.21%)	

^†^This factor was considered as one of the covariates in the statistical study.

### Data acquisition and preprocessing

The MRI scans were preoperatively performed using a Sie mens Prisma 3.0 T scanner in all subjects. We utilized an echo planar imaging (EPI) technique specifically optimized for diffusion MRI, facilitating the subsequent voxel-level modeling necessary for advanced diffusion MRI preprocessing. The EPI parameters consisted of a field of view measuring 1,760 mm × 1,760 mm, a flip angle (FA) of 90°, and b-values of 1,000 and 2,000 s/mm^2^, each with 30 diffusion directions. The echo time (TE) was 64 ms, the repetition time (TR) was 2,900 ms, with each image slice having a thickness of 2.5 mm and an in-plane spatial resolution of 2.5 mm × 2.5 mm, indicating isotropic voxels of 2.5 mm^3^. Preoperative T1-weighted magnetization-prepared rapid gradient-echo (MPRAGE) sequence was utilized with the following parameters: FA of 9°, TR of 1,600 ms, TE of 2.98 ms, inversion time of 900 ms, a matrix size of 256 × 248, slice thickness of 1 mm, and field of view (FOV) of 220 mm^2^ × 220 mm^2^. Additionally, the T2-weighted space dark-fluid sequence was used, with parameters including a FA of 120°, TR of 5,000 ms, TE of 581 ms, inversion time of 1,600 ms, a matrix size of 256 × 256, slice thickness of 1 mm, and FOV of 220 mm^2^ × 220 mm^2^. The eddy tool from FSL (FMRIB Software Library, version 6.0.4)^1^ was used to correct both eddy current distortions and participant movements in diffusion MRI data (Andersson and Sotiropoulos, 2016). Additionally, the diffusion imaging data underwent normalization through q-space diffeomorphic reconstruction (QSDR). The dispersion data from each participant was reassembled in a universal stereotaxic space, which maintains the conservation of diffusion spins after a non-linear spatial transformation as outlined by Yeh and Tseng (2011). Concurrently, the spin distribution function (SDF) measures the concentration or volume of diffused water in every direction inside a specific voxel (Yeh et al., 2016b).

### Differential tractography

**FIGURE 1:**
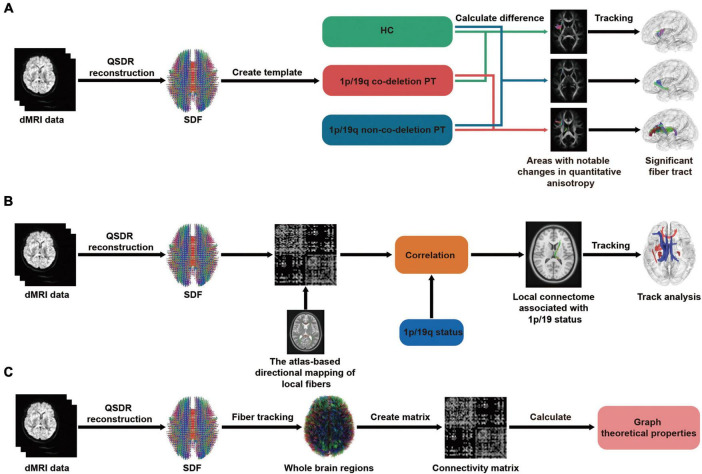
Flowchart of differential, correlational tractography, and graph theoretical analysis. In all three analyses, a consistent step is the transformation of dMRI data, whether from patients or healthy controls (HC), into a standardized format using q-space diffeomorphic reconstruction (QSDR). Additionally, the spin distribution function (SDF) is employed to determine the quantity or concentration of water diffusion in different orientations within a particular voxel. **(A)** Differential tractography: dMRI data are aggregated to form group averages, which then facilitates pairwise comparisons among the 1p/19q co-deleted, non-co-deleted, and HC groups. This process culminates in displaying results with significant differences. **(B)** Correlational tractography: The SDF are sampled and structured into a local connectome matrix. The term “local connectome” refers to the degree of connectivity between adjacent voxels within a white matter fascicle, defined by the density of diffusing spins. The association of this matrix with the 1p/19q status is adjusted for factors like age, gender, tumor volume, and grades, ultimately leading to the identification of fiber components with significant correlations. **(C)** Graph theoretical analysis: Using deterministic fiber tracking and brain segmentation, a connectivity matrix was constructed. Subsequently, both global and local properties derived from graph theory were calculated. These properties were then used for comparative analysis among the 1p/19q co-deleted, non-co-deleted, and HC groups.

The flowchart of differential tractography analysis was demonstrated in Figure 1A. Group-average templates were created by computing the mean SDF values for the 1p/19q co-deletion patients, 1p/19q non-co-deletion patients, and HC using DSI Studio for comparison. These average templates were then utilized to investigate the characteristic brain connections and their variations between the groups. The differential tractogram was generated by positioning a total of 5,000,000 seed points within the white matter. The angular threshold was chosen randomly between 15 and 90 degrees. A step size of 0.5 to 1.5 voxel was set, and the anisotropy threshold was determined automatically by DSI Studio, based on the quality assessment during whole brain fiber tracking. To assess possible alterations in the fiber tracts, differential tractography was executed using various QA change thresholds (20, 30, and 40%) and fiber length thresholds (20, 30, and 40 mm). Any tracks shorter than the set length thresholds and tracks with changes less than the defined QA change thresholds were omitted. A similar method has been detailed thoroughly in a previous study (Yeh et al., 2019). To control the family-wise error rate (FWER) of our *p*-values, we applied the Bonferroni procedure, which adjusts the *p*-values to account for multiple comparisons.

### Correlational tractography

As shown in the flowchart Figure 1B, the diffusion data from all patients was reconstructed in a standardized space. Subsequently, the density of the diffusing spins was sampled according to local fiber directions from a common atlas, facilitating the creation of a local connectome matrix and the estimation of the local connectome. 2.5 T thresholds was employed to map different levels of correlation between the fiber tracts and the 1p/19q co-deletion while controlling for age, sex, tumor volume, and grades through a deterministic fiber tracking algorithm. The QA-values were normalized, and the tracts were refined using topology-informed pruning with 16 iterations. To estimate the false discovery rate, a total of 4,000 randomized permutations were applied to the group label to generate the null distribution of the tract length. Permutation testing allows for estimating and correcting the false discovery rate (FDR) of Type-I error inflation due to multiple comparisons. The FDR, directly estimated from the ratio between total findings and false positive findings, is used to reject the null hypotheses and identify tracks with significant FDR (Yeh et al., 2016a). This approach directly estimates the FDR value, providing a more intuitive measure of significance, which is why we report the FDR value rather than FDR-corrected *p*-values.

### Graph theoretical analysis

We contrasted the local and global properties among patients with 1p/19q co-deletion, those without 1p/19q co-deletion, and HCs. Figure 1C displayed the flowchart of the graph theoretical analysis, whole-brain deterministic tractography was carried out in DSI Studio following the reconstruction process, which is in line with the procedure described above. Restricted diffusion was quantified using restricted diffusion imaging (Yeh et al., 2017). We used a deterministic fiber tracking algorithm enhanced with augmented tracking strategies for better reproducibility (Yeh et al., 2013; Yeh, 2020). Spatial normalization was performed to align the built-in FreeSurfer’s Desikan-Killiany-Tourville (DKT) cortical atlas, encompassing 62 cortical regions of interest, with the subject’s diffusion data. The software includes a specialized nonlinear registration tool designed to align diffusion MRI data with structural MRI data. We built a structural connectome for each participant using a connectivity threshold of 0.001. This involved creating a connectivity matrix by counting the intersecting tracks, leading to a distinct matrix for each individual that represented their brain’s structural connectome. Structural connectivity was then evaluated using graph properties in DSI-Studio software. For each global and local graph variable, we employed analysis of covariance (ANCOVA) to evaluate differences between three groups: the 1p/19q co-deletion group, the 1p/19q non-co-deletion group, and a HC group. This analysis allowed us to discern any significant differences across the three groups, after controlling for potential confounding effects of age and sex. In a separate analysis focused on tumor groups, we examined the differences between the 1p/19q co-deletion and 1p/19q non-co-deletion groups while controlling for age, sex, tumor volume, and grades. We adjusted the resulting FDR-corrected *p*-values using the Benjamini-Hochberg correction method. Importantly, this correction process involved collectively adjusting all *p*-values derived from the comprehensive comparison among the three groups, while *p*-values obtained from the analyses focusing specifically on tumor group comparisons were adjusted separately.

### Statistics

Chi-square test was employed to analyze categorical variables among the patients and HC. After correction, *p*-values that fell below 0.05 were deemed statistically significant. *P*-values represent uncorrected *p*-values. In this study, effect sizes for partial correlations or ANCOVA statistical methods were calculated using Cohen’s *f*^2^. Effect sizes for *t*-tests in differential tractography were calculated using Cohen’s d, and for Wilcoxon tests, Cliff’s Delta was used. Effect sizes are reported in the range 0.0 to 1.0. For our statistical analysis, we used R version 4.2.2 for visualization and statistical calculation, alongside Python 3.11.3, employing “pandas” and “statsmodels” for statistical testing.

## Results

### Differential tractography

**FIGURE 2:**
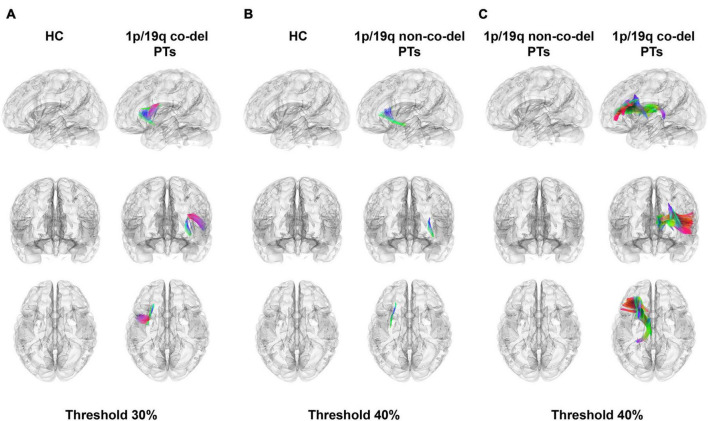
Differential tractography analysis. Color-coded fibers illustrate different tract directions. **(A)** The highlighted panel shows fiber tracts in the healthy control (HC) group with higher quantitative anisotropy (QA) values compared to patients with 1p/19q co-deletion. Notable tracts include the left frontal aslant tract (FAT), inferior fronto-occipital fasciculus (IFOF), arcuate fasciculus (AF), superior corticostriatal tract (SCT), superior thalamic radiation (STR), superior longitudinal fasciculus 3 (SLF 3), superior longitudinal fasciculus 2 (SLF 2), anterior corticostriatal tract (ACT), corticobulbar tract (CT), and uncinate fasciculus (UF). The total volume of these tracts is 4,783.20 mm^3^ (FWER-corrected *p* = 1.827 × 10^–5^, Cohen’s *d* = –1.938), with a threshold set at 30%. **(B)** This panel displays fiber tracts in patients without 1p/19q co-deletion, revealing lower QA values compared to the HC group. Notable tracts include sections of the left IFOF, UF, and SCT, with a total volume of 1,664.06 mm^3^ (FWER-corrected *p* = 1.878 × 10^–5^, Cohen’s *d* = –1.686). The threshold for depiction is set at 40%. **(C)** The contrast between patients with 1p/19q co-deletion and those without highlights substantially lower QA values in specific tracts, including the left ATR, STR, fornix, ACT, dentatorubrothalamic tract, cingulum parahippocampal, and reticular tract. These tracts account for a total volume of 18,484.40 mm^3^ (uncorrected *p* = 0.037, FWER-corrected *p* = 0.111, Cliff’s Delta = –0.441), and the threshold for visualization is set at 40%.

Figure 2 showcased the results of a differential analysis on QA between the HC group, the group with 1p/19q co-deletion, and the group without this co-deletion. The fibers’ tracts in the figure were color-coded, each color signifying a different direction of the tracts. The analysis results were derived with certain conditions in place: a length threshold of 30 mm, and a change threshold within a 30–40% decrease of anisotropy.

Figure 2A illustrated the fiber tracts in the HC group that had higher QA values compared to the patients with 1p/19q co-deletion. These tracts included the left frontal aslant tract (FAT), inferior fronto-occipital fasciculus (IFOF), arcuate fasciculus (AF), superior corticostriatal tract (SCT), superior thalamic radiation (STR), superior longitudinal fasciculus 3 (SLF 3), superior longitudinal fasciculus 2 (SLF 2), anterior corticostriatal tract (ACT), corticobulbar tract (CT), and uncinate fasciculus (UF). The total volume of these tracts was 4,783.20 mm^3^ (FWER-corrected *p* = 1.827 × 10^–5^, Cohen’s *d* = −1.938). Figure 2B depicted fiber tracts in patients without 1p/19q co-deletion having lower QA values compared to the HC group. These tracts included sections of the left IFOF, UF, and SCT, amounting to a total volume of 1,664.06 mm^3^ (FWER-corrected *p* = 1.878 × 10^–5^, Cohen’s *d* = −1.686). The threshold indicated below the figure revealed that the disparity in these three types of fiber tracts between the two groups surpassed 40%. In a similar vein, when compared to patients without 1p/19q co-deletion in part c, patients with 1p/19q co-deletion exhibited considerably lower QA values in the left anterior thalamic radiation (ATR), STR, fornix, ACT, dentatorubrothalamic tract, cingulum parahippocampal, and reticular tract. These tracts had a total volume of 18,484.40 mm^3^ (uncorrected *p* = 0.037, FWER-corrected *p* = 0.111, Cliff’s Delta = −0.441), with the corresponding threshold set at 40%. The detailed results were shown in Supplementary Table 1.

### Correlational tractography

**FIGURE 3:**
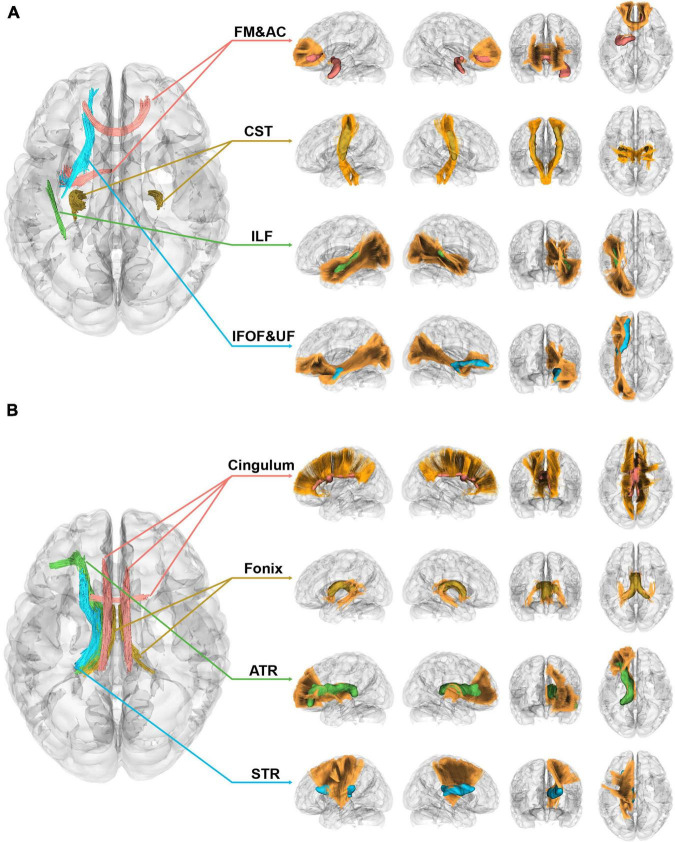
Correlational tractography of left insular gliomas with the 1p/19q co-deletion. **(A)** This section depicts fiber segments that display a significant positive correlation with the 1p/19q co-deletion in patients with left insular gliomas (FDR = 4.29 × 10^–4^, Cohen’s *f*^2^ = 0.103). **(B)** The segment of the figure showcases fiber components that exhibit a significant negative correlation with the 1p/19q co-deletion in patients with left insular gliomas (FDR = 4.72 × 10^–5^, Cohen’s *f*^2^ = 1.13 × 10^–3^).

Figure 3 displayed the correlational analysis outcomes of the 1p/19q co-deletion molecular marker for left insular gliomas, with variables such as age, sex, tumor volume, and grades taken into account. Parts A and B of the figure, respectively, depicted sections with significant positive and negative correlation results. In the left section of both parts, the fiber segments showcasing significant differences were shown axially and were represented by different colors. On the right, the significant sections of the fiber tracts were portrayed in their respective locations within the tracts, with corresponding color codes.

In patients with insular gliomas, the QA values of the forceps minor were found to have a positive correlation with the 1p/19q co-deletion (FDR = 4.29 × 10^–4^, Cohen’s *f*^2^ = 0.103). Remarkable segments, as seen in Figure 3A, were located in the anterior part of the IFOF, the junction of the UF, the anterior-middle part of the ILF, the middle-upper section of the CST, and the middle-lower segment of the forceps minor (FDR = 4.29 × 10^–4^, Cohen’s *f*^2^ = 0.103). On the contrary, Figure 3B demonstrated that the QA values of the ipsilateral ATR, STR, and bilateral fornix and cingulum were inversely related to the 1p/19q co-deletion in patients with left insular gliomas (FDR = 4.72 × 10^–5^, Cohen’s *f*^2^ = 1.13 × 10^–3^). Of these significant fiber tract segments, the fornix constituted the majority, followed by the lower sections of the ATR and STR. The smallest proportion was attributed to the cingulum, which incorporated bilateral parolfactory, parietal, and parahippocampal components, predominantly on the left side. Detailed effect sizes, statistical power, and *p*-values can be found in Supplementary Table 2.

### Graph theoretical analysis

**FIGURE 4:**
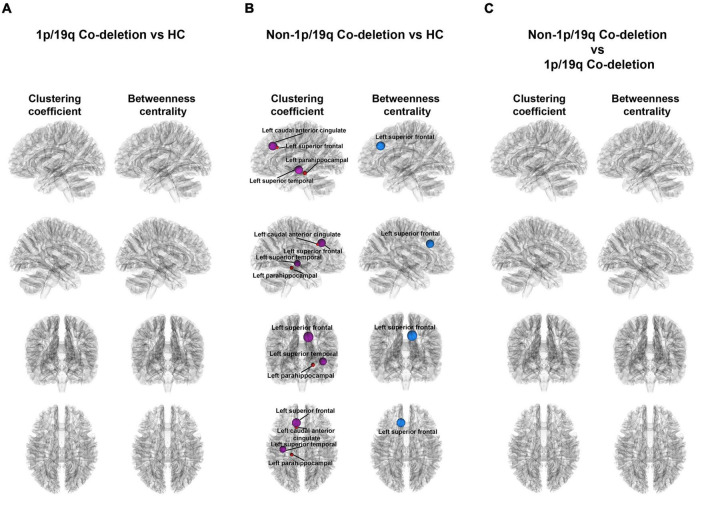
Visual representation of local connectivity variations among different groups. **(A)** There were no significant findings among patients with 1p/19q co-deletion and the HC group, while taking into consideration age and sex factors. **(B)** The diagram emphasizes the substantial discrepancies in local structural connectivity among patients without 1p/19q co-deletion and the HC group (FDR-corrected *p* < 0.05), when accounting for age and sex influences. The blue and red orbs in this context represent nodes with a decrease and increase in local properties, respectively, when compared to the HC group, for patients without 1p/19q co-deletion. **(C)** The illustrated figure brings into focus the variations on binary clustering coefficient and betweenness centrality between patients with and without 1p/19q co-deletion.

Figure 4 illustrated the variations in local properties among the HC, 1p/19q co-deletion, and 1p/19q non-co-deletion groups. The nodes, displayed from top to bottom, corresponded to the left sagittal, right sagittal, anterior coronal, and superior axial views. Every node in the figure symbolized a statistically significant node. Node colors indicated variations in local properties, with red and purple signifying increases and blue showing decreases in pairwise comparisons. There were no significant findings between the HC and 1p/19q co-deletion groups (Figure 4A). Figure 4B showed that patients with 1p/19q non-co-deletion had a significantly higher binary clustering coefficient in the left anterior caudal cingulate (FDR-corrected *p* = 0.026, Cohen’s *f*^2^ = 0.174), parahippocampal (FDR-corrected *p* = 0.041, Cohen’s *f*^2^ = 0.134), left superior frontal (FDR-corrected *p* = 0.002, Cohen’s *f*^2^ = 0.212), superior temporal (FDR-corrected *p* = 0.034, Cohen’s *f*^2^ = 0.160) compared to the HC group, and the corresponding nodes are colored in red or purple in the figure. In terms of binary betweenness centrality, the left superior frontal (FDR-corrected *p* = 0.001, Cohen’s *f*^2^ = 0.429) had significantly lower values in the 1p/19q non-co-deletion group (shown as red or purple nodes in the figure) than HC. Figure 4C showed, after controlling for age, sex, tumor volume, and grades covariates, that compared to patients with 1p/19q co-deletion, the non-1p/19q codeletion group had no significant results in binary clustering coefficient and betweenness centrality, as detailed in Supplementary Tables 3, 4, respectively.

As shown in Table 2, after accounting for sex and age variables, we found no significant disparities in the global properties among the HC, 1p/19q co-deletion, and 1p/19q non-co-deletion groups. When we controlled for sex, age, tumor volume, and grade variables and compared the 1p/19q co-deletion and 1p/19q non-co-deletion groups, we discovered some significant differences before collective correction. The 1p/19q non-co-deletion group showed significant increases in the weighted average clustering coefficient (uncorrected *p* = 0.047, FDR-corrected *p* = 0.640, Cohen’s *f*^2^ = 0.107), weighted transitivity (uncorrected *p* = 0.030, FDR-corrected *p* = 0.640, Cohen’s *f*^2^ = 0.125), weighted path length (*p* = 0.048, FDR-corrected *p* = 0.640, Cohen’s *f*^2^ = 0.133), weighted small worldness (uncorrected *p* = 0.018, FDR-corrected *p* = 0.640, Cohen’s *f*^2^ = 0.132), and weighted global efficiency (uncorrected *p* = 0.017, FDR-corrected *p* = 0.640, Cohen’s *f*^2^ = 0.177). However, these results did not pass the correction. In the binary average clustering coefficient, small worldness, rich club k10, k15, and k20, as well as in weighted rich club k10, k20, and k25, the 1p/19q non-co-deletion group had relatively higher values (Table 2). Furthermore, statistical power for both global and local metrics derived from graph theoretical analysis were calculated using ANCOVA and are documented in Supplementary Table 5.

**TABLE 2:** Comparison of global properties among 1p/19q co-deletion, 1p/19q non-co-deletion, and healthy control groups.

Properties	HC (*n* = 20)	1p/19q CD patients (*n* = 13)	1p/19q NCD patients (*n* = 19)	Un-corrected *p*-value (HC vs. 1p/19q SD patients)	Un-corrected *p*-value (HC vs. 1p/19q NCD patients)	Un-corrected *p*-value (1p/19q CD vs. 1p/19q NCD patients)	FDR-corrected *p*-value (HC vs. 1p/19q CD patients)	FDR-corrected *p*-value (HC vs. 1p/19q NCD patients)	FDR-corrected *p*-value (1p/19q CD vs. 1p/19q NCD patients)	Cohen’s *f*^2^ (HC vs. 1p/19q CD vs. 1p/19q NCD patients)	Uncorrected *p*-value (1p/19q CD vs. 1p/19q NCD patients), controlling for age, sex, tumor volume, and grades	FDR-corrected *p*-value (1p/19q CD vs. 1p/19q NCD), controlling for age, sex, tumor volume, and grades	Cohen’s *f*^2^ (1p/19q CD vs. 1p/19q NCD patients), controlling for age, sex, tumor volume, and grades
Density	0.389 ± 0.044	0.394 ± 0.048	0.408 ± 0.040	0.940	0.387	0.700	1.000	1.000	1.000	0.043	0.367	0.692	0.028
Clustering coff (B)	0.690 ± 0.013	0.690 ± 0.020	0.698 ± 0.021	1.000	0.301	0.416	1.000	1.000	1.000	0.054	0.285	0.640	0.041
Clustering coff (W)	0.023 ± 0.005	0.023 ± 0.003	0.025 ± 0.004	0.996	0.269	0.338	1.000	1.000	1.000	0.073	0.047*	0.640	0.107
Transitivity (B)	0.088 ± 0.031	0.097 ± 0.031	0.111 ± 0.022	0.620	0.051	0.490	1.000	1.000	1.000	0.134	0.293	0.692	0.067
Transitivity (W)	0.019 ± 0.005	0.019 ± 0.003	0.022 ± 0.004	0.994	0.167	0.315	1.000	1.000	1.000	0.094	0.030*	0.640	0.125
Path Length (B)	1.654 ± 0.097	1.645 ± 0.083	1.622 ± 0.054	0.958	0.460	0.734	1.000	1.000	1.000	0.034	0.458	0.692	0.031
Path Length (W)	21.900 ± 9.389	20.229 ± 4.870	17.225 ± 3.659	0.836	0.108	0.456	1.000	1.000	1.000	0.098	0.048*	0.640	0.133
Small-Worldness (B)	0.418 ± 0.025	0.420 ± 0.021	0.431 ± 0.023	0.977	0.214	0.427	1.000	1.000	1.000	0.070	0.206	0.640	0.065
Small-Worldness (W)	0.001 ± 0.0008	0.001 ± 0.0005	0.002 ± 0.0005	0.864	0.500	0.319	1.000	1.000	1.000	0.057	0.018*	0.640	0.132
Global efficiency (B)	0.677 ± 0.029	0.680 ± 0.029	0.689 ± 0.022	0.950	0.412	0.706	1.000	1.000	1.000	0.040	0.404	0.692	0.030
Global efficiency (W)	0.072 ± 0.026	0.070 ± 0.015	0.083 ± 0.015	0.947	0.314	0.274	1.000	1.000	1.000	0.078	0.017*	0.640	0.177
Diameter of graph (B)	3.050 ± 0.224	3.077 ± 0.277	3.000 ± 0.000	0.757	0.610	0.300	1.000	1.000	1.000	0.027	0.119	0.640	0.049
Diameter of graph (W)	77.815 ± 93.435	55.954 ± 20.964	52.662 ± 21.356	0.755	0.408	0.913	1.000	1.000	1.000	0.039	0.515	0.713	0.006
Radius of graph (B)	2.000 ± 0.000	2.000 ± 0.000	2.000 ± 0.000	0.514	0.627	0.957	1.000	1.000	1.000	0.032	0.375	0.970	0.023
Radius of graph (W)	49.780 ± 84.040	30.795 ± 10.475	29.386 ± 13.939	0.773	0.456	0.930	1.000	1.000	1.000	0.035	0.411	0.692	0.003
Assortativity coff (B)	−0.082 ± 0.030	−0.081 ± 0.029	−0.064 ± 0.037	0.995	0.217	0.372	1.000	1.000	1.000	0.077	0.264	0.692	0.067
Assortativity coff (W)	−0.076 ± 0.026	−0.046 ± 0.057	−0.056 ± 0.031	0.080	0.308	0.658	1.000	1.000	1.000	0.113	0.267	0.692	0.015
Rich club K5 (B)	0.391 ± 0.043	0.397 ± 0.045	0.409 ± 0.039	0.922	0.450	0.785	1.000	1.000	1.000	0.037	0.437	0.692	0.021
Rich club K10 (B)	0.430 ± 0.033	0.425 ± 0.042	0.436 ± 0.035	0.940	0.905	0.772	1.000	1.000	1.000	0.013	0.487	0.777	0.019
Rich club K15 (B)	0.500 ± 0.030	0.490 ± 0.025	0.504 ± 0.028	0.688	0.932	0.516	1.000	1.000	1.000	0.040	0.235	0.640	0.069
Rich club K20 (B)	0.610 ± 0.028	0.618 ± 0.033	0.611 ± 0.037	0.755	0.996	0.812	1.000	1.000	1.000	0.011	0.295	0.692	0.010
Rich club K25 (B)	0.742 ± 0.066	0.766 ± 0.077	0.744 ± 0.047	0.691	0.995	0.756	1.000	1.000	1.000	0.027	0.347	0.692	0.034
Rich club K5 (W)	0.999 ± 0.002	0.999 ± 0.003	1.000 ± 0.0002	0.630	0.267	0.077	1.000	1.000	1.000	0.069	0.064	0.640	0.104
Rich club K10 (W)	0.961 ± 0.027	0.967 ± 0.027	0.977 ± 0.018	0.639	0.161	0.759	1.000	1.000	1.000	0.089	0.543	0.692	0.050
Rich club K15 (W)	0.829 ± 0.069	0.843 ± 0.105	0.853 ± 0.072	0.913	0.633	0.921	1.000	1.000	1.000	0.017	0.729	0.935	0.003
Rich club K20 (W)	0.607 ± 0.102	0.601 ± 0.127	0.649 ± 0.134	1.000	0.572	0.656	1.000	1.000	1.000	0.033	0.312	0.692	0.034
Rich club K25 (W)	0.391 ± 0.106	0.417 ± 0.074	0.438 ± 0.096	0.940	0.387	0.700	1.000	1.000	1.000	0.047	0.305	0.692	0.014

*p < 0.05. B is binary and W is weighted. HC = healthy controls, 1p/19q CD = 1p/19q Co-deletion, 1p/19q NCD = 1p/19q non-co-deletion. Bold values indicate the significant values.

## Discussion

Our study revealed that diffuse gliomas located in the insula of the dominant hemisphere, and featuring a 1p/19q co-deletion (typical of oligodendrogliomas), exhibit pronounced alterations in both structural connectivity and graph-theoretic brain network metrics. The correlational tractography has revealed both positive and negative associations between the 1p/19q co-deletion and the QA values of specific fiber tracts. This correlation deepens understanding of the selective invasion driven by 1p/19q co-deletion. It’s important to highlight that the group without 1p/19q co-deletion shows some peritumoral brain regions, characterized by an increased clustering coefficient and a decrease in betweenness centrality, compared to the group with co-deletion. Moreover, the graph network suggests higher transitivity within the non-co-deletion group, indicative of more efficient information exchange within their neural networks. These observations may have significant implications for our understanding of how the 1p/19q status could affect the architecture of brain networks and, as a result, the cognitive functions in these patients. The results of our study emphasize the complex impacts of gliomas and the inherent genetic makeup on the structural neural network.

A crucial finding of this study was that patients with insular gliomas exhibiting the 1p/19q co-deletion tend to selectively infiltrate and disrupt the deep and medial white matter fiber tracts. Prior studies have proposed that diffuse gliomas with different molecular phenotypes may exhibit preferences for distinct locations (Laigle-Donadey et al., 2004; Metellus et al., 2010; Latini et al., 2020). For instance, Laigle-Donadey et al. (2004) have demonstrated that oligodendrogliomas with a chr 1p loss are more likely to be located in the frontal lobes. Another study categorizing 102 low-grade patients found that oligodendrogliomas extensively infiltrate white matter networks, such as association, commissural, and projection pathways, with a notable presence between the basal ganglia and deep and mesial regions of both frontal lobes (Latini et al., 2020). This study’s correlational tractography of insular gliomas corroborated these findings, with alterations in QA values indicating changes in the integrity of fibers surrounding the glioma, potentially serving as objective imaging markers (Celtikci et al., 2018). QA values of ipsilateral ATR, STR, and primarily left-sided fornix and cingulum were found to be inversely related to the co-deletion status, suggesting a decrease in QA values for these fiber tracts. Conversely, the 1p/19q co-deletion positively correlated with the QA values of ipsilateral association fibers IFOF, UF, and ILF, along with bilateral projection fibers CST and the commissural fiber AC. The integrity of these tracts of insular gliomas in the 1p/19q co-deletion group was relatively high, indicating less impact from tumor infiltration and disruption. The negative affects relatively limited magnitude highlights that such adaptations are far less effective and extensive compared to the pervasive positive effects caused by of 1p/19q co-deletion. Compared to the healthy control group, the QA values of SCT, STR, and CT, which are nearer to the medial side, demonstrated a decrease in 1p/19q co-deletion patients. However, in the 1p/19q non-co-deletion patients, only the QA value of the medial SCT was found to decrease. A comparison of the QA values of deep medial fibers such as ATR, STR, fornix, ACT, dentatorubrothalamic tract, cingulum, and reticular tract showed lower values in the 1p/19q co-deletion group as compared to the 1p/19q non-co-deletion group. The specific mechanisms and reasons for oligodendrogliomas selectively compromising the integrity of certain white matter tracts remain elusive. One hypothesis is that this phenomenon may be driven by the unique growth and migratory characteristics of oligodendroglioma cells. Research indicates that the infiltration patterns of gliomas mirror cellular migration processes seen during brain development, favoring the navigation of white matter tracts from the globus pallidus internus toward the cortical regions (Marin and Rubenstein, 2003; Tripathi et al., 2011; Zhan et al., 2017; Skjulsvik et al., 2020). As tumor cells infiltrate the deep subcortical white matter, they tend to follow paths akin to their original locations, demonstrating a consistent invasion pattern directed toward the central core and basal ganglia (Painter and Hillen, 2013; Fathallah-Shaykh et al., 2019). Our findings elucidate the characteristic invasion patterns of insular oligodendrogliomas. In addition, a recent study has shown that AF and IFOF connecting the anterior and posterior parts of the perisylvian areas, which are relevant for language function (Fekonja et al., 2021). Moreover, research by Shams et al. (2022) using diffusion MRI and machine learning suggests that microstructural changes in white matter effectively predict glioma-induced functional deficits. Therefore, our findings might suggest that patients with 1p/19q co-deletion could potentially have a lesser impact on language functions. The insights gained from our research into the invasion pattern of insular oligodendrogliomas could be particularly beneficial in monitoring management and guiding surgical planning to minimize potential damage to critical white matter tracts and their functions.

It is known that oligodendrogliomas demonstrate less aggressive traits compared to astrocytomas (Donovan and Lassman, 2019). Additionally, compared to oligodendrogliomas, astrocytoma could utilize ultra-long membrane protrusions called tumor microtubes to facilitate invasion, proliferation, and intercellular communication (Osswald et al., 2015). Our investigation revealed that only the 1p/19q non-co-deletion group (astrocytoma) had peritumoral brain regions with an increased clustering coefficient and decreased betweenness centrality compared to the HC group. The corresponding substantial effect sizes underscore the strong and potentially clinically relevant relationships between the local metrics and 1p/19q non-co-deletion status. Although differences between the 1p/19q co-deletion group and HCs were not significant after FDR correction, the clustering coefficient was higher in the 1p/19q co-deletion group compared to HCs, yet lower than in the 1p/19q non-co-deletion group. Astrocytomas, known for their extensive impact and destructiveness, may exploit the intrinsic neuroplasticity near the tumor site. The superior frontal gyrus is a crucial component of the dorsolateral prefrontal cortex (DLPFC), which is intricately connected to almost every cortical and subcortical structure (Szczepanski and Knight, 2014). The comprehensive reductions in betweenness centrality in superior frontal may signify a decrement in the pathways for information transmission within the network. On the other hand, an escalation in the binary clustering coefficient may be indicative of an increased density of neuronal connections within these regions. These changes, potentially indicative of astrocytomas, could be a compensatory mechanism to mitigate deficiencies in long-distance communication, achieved by prioritizing and amplifying local communication within clusters (Watts and Strogatz, 1998; Park et al., 2016; Semmel et al., 2022). In addition, although there is no significant difference in tumor volume between the 1p/19q co-deletion and non-co-deletion groups, we cannot completely rule out the possibility that the larger tumors in the non-co-deletion group extensively invade surrounding brain regions, leading to increased clustering coefficient and decreased betweenness centrality in these areas.

This study indicates that glioma patients with co-deletion tend to have lower global transmission efficiency. Although these findings did not withstand correction for multiple comparisons, they were supported by a high statistical power. Localized shifts in brain network operations can induce significant ripple effects across the entire global network. This study undertook an analysis of a multitude of global properties. Among these, the average clustering coefficient, in contrast to the clustering coefficient, serves as a global property that evaluates the level of network segregation. It does so by quantifying the extent of clustered connections that surround individual nodes. Transitivity is determined based on the ratio of triangles to triplets of nodes, serving as an alternative to the clustering coefficient (Semmel et al., 2022). Without controlling for additional variables like tumor volume and grades, our study found no significant differences in the average weight clustering coefficient and weighted transitivity among the 1p/19q co-deletion, non-co-deletion, and the HC groups. It’s worth noting that the volume and proliferation rate of gliomas can also influence the graph theoretical network and should be considered during comparative analysis (Semmel et al., 2022). Changes in global integration properties, such as global efficiency, small-worldness, and characteristic path length, remain contested when comparing tumor patients among themselves or against HC (van Dellen et al., 2012; Park et al., 2016; De Baene et al., 2017; Aerts et al., 2018). Small-world patterns merge “high levels of local clustering among nodes of a network (forming families or cliques) and short paths that globally connect all nodes of the network,” implying that nodes are “linked through relatively few intermediary steps.” Characteristic path length is the average count of path segments required to link one node to another, with fewer steps indicating higher efficiency. Global efficiency is computed as the harmonic inverse of the characteristic path length (Bullmore and Sporns, 2009). Research by Kesler and colleagues found that less invasive IDH1 mutant gliomas had significantly higher global efficiency in brain networks than wild-type tumors (Kesler et al., 2017). This study found that 1p/19q non-co-deletion patients showed increases in weighted small worldness and global efficiency, and a decrease in path length compared to 1p/19q co-deletion patients, although these findings did not withstand correction. Similarly, two studies showed that, when using magnetoencephalography (MEG), LGG patients exhibited lower intermodular connectivity compared to HGG patients (van Dellen et al., 2012). Thus, we theorize that gliomas, particularly those 1p/19q co-deletion, significantly damage essential deep commissural fibers, such as the cingulum, an important hub in the structural network (Li et al., 2013; van den Heuvel and Sporns, 2013). This disruption may result in a decrease in the overall transmission efficiency and local connectivity of the brain network. Moreover, the presence of an insular astrocytoma may catalyze a “network economy” strategy, tactfully maintaining a balance between wiring cost constraints, spatial and metabolic resources, and the imperatives to optimize network performance. The escalation in weighted small-worldness might enhance low-cost specialization and integration, both associated with improved efficiency. There is conjecture that the incursion of aggressive astrocytomas might exacerbate disruptions to the brain’s connectome, prompting compensatory improvements in transmission efficiency and local connectivity. These adjustments can be interpreted as the brain’s strategic response to maintain functional coherence and performance despite the pathological disturbances instigated by the tumor. The “network economy” strategy, which enhances local and integrative connectivity to preserve network functionality and adapt to disease effects, may inadvertently make these regions more susceptible to glioma. Furthermore, this strategy could potentially stimulate tumor growth and spread by cultivating a hyperconnected environment, thus amplifying resource availability for the glioma (Mandal et al., 2020; Douw et al., 2023). This possible setback underscores the need for a personalized approach when comprehending and addressing gliomas.

Conversely, when examining binary network metrics, we didn’t identify significant differences between the 1p/19q co-deletion and non-co-deletion groups in clustering coefficient, transitivity, path length, small-worldness, and global efficiency. This suggests that the impact of glioma on brain networks may be more pronounced when considering the strength and quality of neuronal connections, rather than their mere presence or absence. The observed significant differences prior to global correction point to a differential impact of 1p/19q co-deletion status on the nuanced aspects of network topology, potentially reflecting underlying biological differences in tumor pathology and its effects on brain connectivity. However, the absence of significant differences in both unweighted and weighted network parameters after global correction, along with the non-significant variations in other global network characteristics like the graph’s diameter, radius, and assortativity across all groups, prompts a cautious interpretation of these findings. This highlights the complexity of brain network adaptation to glioma and suggests that the brain may employ robust mechanisms to preserve network functionality, even in the face of pathological changes. The consistent lack of significant differences in several key network metrics across patient and control groups, regardless of 1p/19q deletion status, emphasizes the need for further research to unravel the compensatory and adaptive strategies employed by brain networks in response to glioma.

The significant differences observed in weighted network metrics between 1p/19q co-deletion and non-co-deletion groups, although not surviving global correction, alongside the general lack of significant differences in other network metrics, reflect the complex interplay between glioma pathology and brain network dynamics. This complexity calls for a deeper exploration of how gliomas, with varying molecular characteristics, affect the brain’s structural and functional connectivity, furthering our understanding of the neurobiological implications of these tumors.

### Limitations

This study faces several limitations. Our retrospective design could lead to selection bias, affecting the generalizability of our findings. The cross-sectional nature also limits our ability to observe glioma progression or treatment response over time. Particularly, the small sample size reduces our ability to detect subtle differences, a concern amplified by the variability and complexity of dMRI and tractography techniques. Our diffusion MRI protocol lacked reverse phase-encoding, which might impact data quality and tractography accuracy. Despite the sophistication of the tractography algorithm used, it may not fully capture the complexities of white matter structures. Additionally, we excluded right hemisphere insular gliomas (24 patients) due to the extreme imbalance. This significant disparity could bias our results and potentially lead to inaccurate conclusions. Therefore, we focused solely on left insular gliomas to maintain the integrity of our findings. This exclusion is a specific limitation, highlighting a potential bias in our study and indicating that our results may be more representative of left insular gliomas. Future research should address these issues with larger, longitudinal designs, diverse tractography algorithms, reverse phase-encoding, multi-modal imaging, and more comprehensive patient data. Ensuring the inclusion of effect sizes and conducting thorough power analyses will be crucial for a clearer and more informative presentation of outcomes.

## Conclusion

This study revealed that in patients with insular glioma, the presence of 1p/19q co-deletion significantly impacts structural connectivity. Tumors with this co-deletion penetrate and disrupt midline white matter hubs more than those without it. Patients with non-1p/19q co-deletion showed more significant effects on their brain networks than those with 1p/19q co-deletion. This research enhances our understanding of how tumor molecular markers affect brain networks and informs strategies for tumor monitoring and function preservation in clinical practice.

## Data availability statement

The raw data supporting the conclusions of this article will be made available upon request by contacting the corresponding author at xiejianw@yeah.net.

## Ethics statement

The studies involving humans were approved by the Ethics Community of Beijing Tiantan Hospital, Capital Medical University (KY 2020-146-02). The studies were conducted in accordance with the local legislation and institutional requirements. The participants provided their written informed consent to participate in this study.

## Author contributions

Z-cY: Data curation, Methodology, Software, Visualization, Writing – original draft, Writing – review & editing. B-wX: Data curation, Writing – review & editing. X-yS: Data curation, Writing – review & editing. C-dY: Data curation, Investigation, Writing – review & editing. F-cY: Conceptualization, Formal Analysis, Methodology, Software, Supervision, Visualization, Writing – review & editing. GL: Data curation, Writing – review & editing. Z-hD: Project administration, Supervision, Writing – review & editing. S-jS: Investigation, Project administration, Supervision, Validation, Writing – review & editing. Z-gH: Conceptualization, Investigation, Project administration, Resources, Supervision, Writing – review & editing. JX: Conceptualization, Funding acquisition, Investigation, Project administration, Resources, Supervision, Writing – review & editing.

## Footnotes

1
https://fsl.fmrib.ox.ac.uk/fsl/fslwiki/
