# Supplementary material for: Connectomic insights into the impact of 1p/19q co-deletion in dominant hemisphere insular glioma patients
Source: Front Neurosci. 2024 Jul 29;18:1283518. doi: 10.3389/fnins.2024.1283518 (PMC11317282; doi:10.3389/fnins.2024.1283518)
Supplement: Supplementary file 1 [file Table_1.docx]

**S-Table 1 Significant fiber tracts, effect sizes, power, and p-values from differential tractography**

| **HC vs 1p/19q co-del PTs** | | | |  |  | **HC vs 1p/19q non-co-del PTs** | | | |  |  | **1p/19q non-co-del vs 1p/19q co-del PTs** | | | |  |
| --- | --- | --- | --- | --- | --- | --- | --- | --- | --- | --- | --- | --- | --- | --- | --- | --- |
| **Significant tracts** | **Cohen's d** | **Power (%)** | **uncorrected p-value** | **FWER-corrected p-value** |  | **Significant tracts** | **Cohen's d** | **Power (%)** | **uncorrected p-value** | **FWER-corrected p-value** |  | **Significant tracts** | **Cliff's Delta** | **Power (%)** | **uncorrected p-value** | **FWER-corrected p-value** |
| Frontal aslant tract | -1.938 | 99.95 | 6.09×10^-6^ | 1.827×10^-5^ |  | Inferior fronto-occipital fasciculus | -1.686 | 99.92 | 6.26×10^-6^ | 1.878×10^-5^ |  | Anterior thalamic radiation | -0.441 | 21.36 | 0.037 | 0.111 |
| Inferior fronto-occipital fasciculus | -1.938 | 99.95 | 6.09×10^-6^ | 1.827×10^-5^ |  | Uncinate fasciculus | -1.686 | 99.92 | 6.26×10^-6^ | 1.878×10^-5^ |  | Superior thalamic radiation | -0.441 | 21.36 | 0.037 | 0.111 |
| Arcuate fasciculus | -1.938 | 99.95 | 6.09×10^-6^ | 1.827×10^-5^ |  | Superior corticostriatal tract | -1.686 | 99.92 | 6.26×10^-6^ | 1.878×10^-5^ |  | Fornix | -0.441 | 21.36 | 0.037 | 0.111 |
| Superior corticostriatal tract | -1.938 | 99.95 | 6.09×10^-6^ | 1.827×10^-5^ |  |  |  |  |  |  |  | Anterior corticostriatal tract | -0.441 | 21.36 | 0.037 | 0.111 |
| Superior thalamic radiation | -1.938 | 99.95 | 6.09×10^-6^ | 1.827×10^-5^ |  |  |  |  |  |  |  | Dentatorubrothalamic tract | -0.441 | 21.36 | 0.037 | 0.111 |
| Superior longitudinal fasciculus 3 | -1.938 | 99.95 | 6.09×10^-6^ | 1.827×10^-5^ |  |  |  |  |  |  |  | Cingulum parahippocampal | -0.441 | 21.36 | 0.037 | 0.111 |
| Superior longitudinal fasciculus 2 | -1.938 | 99.95 | 6.09×10^-6^ | 1.827×10^-5^ |  |  |  |  |  |  |  | Reticular tract | -0.441 | 21.36 | 0.037 | 0.111 |
| Anterior corticostriatal tract | -1.938 | 99.95 | 6.09×10^-6^ | 1.827×10^-5^ |  |  |  |  |  |  |  |  |  |  |  |  |
| Corticobulbar tract | -1.938 | 99.95 | 6.09×10^-6^ | 1.827×10^-5^ |  |  |  |  |  |  |  |  |  |  |  |  |
| Uncinate fasciculus | -1.938 | 99.95 | 6.09×10^-6^ | 1.827×10^-5^ |  |  |  |  |  |  |  |  |  |  |  |  |
