# Supplementary material for: Connectomic insights into the impact of 1p/19q co-deletion in dominant hemisphere insular glioma patients
Source: Front Neurosci. 2024 Jul 29;18:1283518. doi: 10.3389/fnins.2024.1283518 (PMC11317282; doi:10.3389/fnins.2024.1283518)
Supplement: Supplementary file 2 [file Table_2.docx]

**S-Table 2 Significant fiber tracts, effect sizes, power, and false discovery rate (FDR) from correlational tractography**

| **Significant tracts** | **Cohen's f^2^** | **Power (%)** | **FDR** |
| --- | --- | --- | --- |
| **Positive correlation** |  |  |  |
| Anterior commissure | 0.103 | 20.13 | 4.29×10^-4^ |
| Corpus callosum forceps minor | 0.103 | 20.13 | 4.29×10^-4^ |
| Corticospinal tract | 0.103 | 20.13 | 4.29×10^-4^ |
| Inferior longitudinal fasciculus | 0.103 | 20.13 | 4.29×10^-4^ |
| Inferior fronto-occipital fasciculus＆Uncinate fasciculus | 0.103 | 20.13 | 4.29×10^-4^ |
| **Negative correlation** |  |  |  |
| Cingulum | 1.13×10^-3^ | 5.12 | 4.72×10^-5^ |
| Fonix | 1.13×10^-3^ | 5.12 | 4.72×10^-5^ |
| Anterior thalamic radiation | 1.13×10^-3^ | 5.12 | 4.72×10^-5^ |
| Superior thalamic radiation | 1.13×10^-3^ | 5.12 | 4.72×10^-5^ |
