# Supplementary material for: Connectomic insights into the impact of 1p/19q co-deletion in dominant hemisphere insular glioma patients
Source: Front Neurosci. 2024 Jul 29;18:1283518. doi: 10.3389/fnins.2024.1283518 (PMC11317282; doi:10.3389/fnins.2024.1283518)
Supplement: Supplementary file 3 [file Table_3.docx]

**S-Table 3 The differences in binary clustering coefficient among healthy control, 1p/19q co-deletion, and 1p/19q non-co-deletion groups**

| **Regions** | **HC (n=20)** | |  | **1p/19q co-deletion patients (n=13)** | |  | **1p/19q non-co-deletion patients (n=19)** | | **FDR-corrected p-value (HC vs 1p/19q co-deletion patients)**, **controlling for age and sex** | **FDR-corrected p-value (HC vs 1p/19q non-co-deletion patients)**, **controlling for age and sex** | **FDR-corrected p-value (1p/19q co-deletion vs 1p/19q non-co-deletion patients)**, **controlling for age and sex** | **Cohen’s f^2^ (HC vs 1p/19q CD vs 1p/19q NCD patients)** | **FDR-corrected (1p/19q co-deletion vs 1p/19q non-co-deletion patients)**, **controlling for age, sex, tumor volume, and grades** | **Cohen’s f^2^ (1p/19q CD vs 1p/19q NCD patients) , controlling for age, sex, tumor volume, and grades** |
| --- | --- | --- | --- | --- | --- | --- | --- | --- | --- | --- | --- | --- | --- | --- |
|  | **Mean** | **SD** |  | **Mean** | **SD** |  | **Mean** | **SD** |  |  |  |  |  |  |
| Left Caudal Anterior Cingulate | 0.724 | 0.101 |  | 0.800 | 0.096 |  | 0.824 | 0.076 | 1.000 | 0.026 * | 1.000 | 0.174 | 0.681 | 0.073 |
| Left Caudal Middle Frontal | 0.855 | 0.086 |  | 0.791 | 0.111 |  | 0.827 | 0.121 | 1.000 | 1.000 | 1.000 | 0.029 | 0.907 | 0.024 |
| Left Cuneus | 0.700 | 0.126 |  | 0.710 | 0.132 |  | 0.700 | 0.117 | 1.000 | 1.000 | 1.000 | 0.002 | 0.946 | 0.002 |
| Left Entorhinal | 0.656 | 0.076 |  | 0.666 | 0.118 |  | 0.725 | 0.090 | 1.000 | 0.992 | 1.000 | 0.034 | 0.907 | 0.021 |
| Left Fusiform | 0.707 | 0.098 |  | 0.741 | 0.120 |  | 0.773 | 0.073 | 1.000 | 1.000 | 1.000 | 0.027 | 0.985 | 0 |
| Left Inferior Parietal | 0.748 | 0.085 |  | 0.700 | 0.098 |  | 0.728 | 0.090 | 1.000 | 1.000 | 1.000 | 0.009 | 0.946 | 0.003 |
| Left Inferior Temporal | 0.603 | 0.091 |  | 0.620 | 0.067 |  | 0.687 | 0.104 | 1.000 | 0.338 | 1.000 | 0.067 | 0.907 | 0.018 |
| Left Isthmus Cingulate | 0.641 | 0.077 |  | 0.654 | 0.048 |  | 0.653 | 0.074 | 1.000 | 1.000 | 1.000 | 0.003 | 0.946 | 0.002 |
| Left Lateral Occipital | 0.728 | 0.087 |  | 0.764 | 0.117 |  | 0.749 | 0.106 | 1.000 | 1.000 | 1.000 | 0.006 | 0.946 | 0.004 |
| Left Lateral Orbitofrontal | 0.592 | 0.059 |  | 0.552 | 0.046 |  | 0.568 | 0.072 | 1.000 | 1.000 | 1.000 | 0.019 | 0.946 | 0.006 |
| Left Lingual | 0.732 | 0.109 |  | 0.713 | 0.065 |  | 0.694 | 0.092 | 1.000 | 1.000 | 1.000 | 0.012 | 0.964 | 0.001 |
| Left Medial Orbitofrontal | 0.641 | 0.073 |  | 0.634 | 0.081 |  | 0.614 | 0.088 | 1.000 | 1.000 | 1.000 | 0.012 | 0.907 | 0.02 |
| Left Middle Temporal | 0.712 | 0.099 |  | 0.699 | 0.117 |  | 0.718 | 0.097 | 1.000 | 1.000 | 1.000 | 0.033 | 0.907 | 0.019 |
| Left Parahippocampal | 0.677 | 0.070 |  | 0.687 | 0.080 |  | 0.781 | 0.101 | 1.000 | 0.041 * | 0.104 | 0.134 | 0.117 | 0.141 |
| Left Paracentral | 0.705 | 0.120 |  | 0.724 | 0.116 |  | 0.737 | 0.071 | 1.000 | 1.000 | 1.000 | 0.008 | 0.946 | 0.002 |
| Left Pars Opercularis | 0.749 | 0.096 |  | 0.735 | 0.129 |  | 0.749 | 0.105 | 1.000 | 1.000 | 1.000 | 0.004 | 0.907 | 0.022 |
| Left Pars Orbitalis | 0.915 | 0.105 |  | 0.867 | 0.121 |  | 0.814 | 0.121 | 1.000 | 0.335 | 1.000 | 0.059 | 0.907 | 0.018 |
| Left Pars Triangularis | 0.749 | 0.081 |  | 0.753 | 0.117 |  | 0.718 | 0.099 | 1.000 | 1.000 | 1.000 | 0.010 | 0.907 | 0.014 |
| Left Pericalcarine | 0.759 | 0.130 |  | 0.789 | 0.131 |  | 0.732 | 0.103 | 1.000 | 1.000 | 1.000 | 0.020 | 0.907 | 0.024 |
| Left Postcentral | 0.606 | 0.052 |  | 0.594 | 0.037 |  | 0.630 | 0.072 | 1.000 | 1.000 | 1.000 | 0.019 | 0.780 | 0.064 |
| Left Posterior Cingulate | 0.690 | 0.073 |  | 0.700 | 0.072 |  | 0.767 | 0.079 | 1.000 | 0.133 | 0.386 | 0.092 | 0.480 | 0.11 |
| Left Precentral | 0.558 | 0.061 |  | 0.574 | 0.058 |  | 0.579 | 0.066 | 1.000 | 1.000 | 1.000 | 0.007 | 0.985 | 0 |
| Left Precuneus | 0.528 | 0.039 |  | 0.531 | 0.048 |  | 0.535 | 0.046 | 1.000 | 1.000 | 1.000 | 0.003 | 0.907 | 0.009 |
| Left Rostral Anterior Cingulate | 0.705 | 0.074 |  | 0.714 | 0.067 |  | 0.712 | 0.064 | 1.000 | 1.000 | 1.000 | 0.001 | 0.907 | 0.004 |
| Left Rostral Middle Frontal | 0.668 | 0.084 |  | 0.711 | 0.119 |  | 0.661 | 0.082 | 1.000 | 1.000 | 1.000 | 0.025 | 0.801 | 0.047 |
| Left Superior Frontal | 0.454 | 0.047 |  | 0.509 | 0.043 |  | 0.545 | 0.062 | 0.335 | 0.002** | 1.000 | 0.212 | 0.801 | 0.055 |
| Left Superior Parietal | 0.573 | 0.083 |  | 0.542 | 0.052 |  | 0.578 | 0.103 | 1.000 | 1.000 | 1.000 | 0.020 | 0.907 | 0.008 |
| Left Superior Temporal | 0.523 | 0.054 |  | 0.546 | 0.070 |  | 0.601 | 0.074 | 1.000 | 0.034 * | 0.190 | 0.160 | 0.423 | 0.157 |
| Left Supramarginal | 0.808 | 0.089 |  | 0.796 | 0.066 |  | 0.829 | 0.102 | 1.000 | 1.000 | 1.000 | 0.003 | 0.907 | 0.013 |
| Left Transverse Temporal | 0.945 | 0.058 |  | 0.908 | 0.116 |  | 0.845 | 0.094 | 1.000 | 0.106 | 1.000 | 0.067 | 0.907 | 0.014 |
| Left Insula | 0.617 | 0.077 |  | 0.719 | 0.126 |  | 0.726 | 0.119 | 0.469 | 0.104 | 1.000 | 0.184 | 0.907 | 0.018 |
| Right Superior Temporal | 0.561 | 0.079 |  | 0.580 | 0.068 |  | 0.546 | 0.040 | 1.000 | 1.000 | 1.000 | 0.012 | 0.801 | 0.028 |
| Right Caudal Anterior Cingulate | 0.724 | 0.097 |  | 0.739 | 0.074 |  | 0.716 | 0.064 | 1.000 | 1.000 | 1.000 | 0.002 | 0.946 | 0.003 |
| Right Caudal Middle Frontal | 0.833 | 0.112 |  | 0.858 | 0.114 |  | 0.837 | 0.125 | 1.000 | 1.000 | 1.000 | 0.004 | 0.985 | 0 |
| Right Cuneus | 0.727 | 0.128 |  | 0.669 | 0.110 |  | 0.684 | 0.108 | 1.000 | 1.000 | 1.000 | 0.021 | 0.946 | 0.01 |
| Right Entorhinal | 0.676 | 0.177 |  | 0.703 | 0.077 |  | 0.686 | 0.104 | 1.000 | 1.000 | 1.000 | 0.001 | 0.985 | 0 |
| Right Fusiform | 0.689 | 0.062 |  | 0.765 | 0.121 |  | 0.774 | 0.087 | 0.344 | 0.207 | 1.000 | 0.095 | 0.964 | 0.001 |
| Right Inferior Parietal | 0.708 | 0.087 |  | 0.669 | 0.087 |  | 0.718 | 0.130 | 1.000 | 1.000 | 1.000 | 0.030 | 0.801 | 0.052 |
| Right Inferior Temporal | 0.580 | 0.099 |  | 0.587 | 0.084 |  | 0.596 | 0.056 | 1.000 | 1.000 | 1.000 | 0.002 | 0.985 | 0 |
| Right Isthmus Cingulate | 0.677 | 0.082 |  | 0.616 | 0.080 |  | 0.655 | 0.054 | 0.338 | 1.000 | 1.000 | 0.039 | 0.907 | 0.013 |
| Right Lateral Occipital | 0.708 | 0.098 |  | 0.695 | 0.098 |  | 0.711 | 0.121 | 1.000 | 1.000 | 1.000 | 0.015 | 0.907 | 0.028 |
| Right Lateral Orbitofrontal | 0.552 | 0.064 |  | 0.537 | 0.069 |  | 0.560 | 0.048 | 1.000 | 1.000 | 1.000 | 0.000 | 0.946 | 0.001 |
| Right Lingual | 0.708 | 0.089 |  | 0.728 | 0.072 |  | 0.737 | 0.084 | 1.000 | 1.000 | 1.000 | 0.011 | 0.985 | 0 |
| Right Medial Orbitofrontal | 0.660 | 0.060 |  | 0.645 | 0.095 |  | 0.616 | 0.056 | 1.000 | 1.000 | 1.000 | 0.025 | 0.907 | 0.013 |
| Right Middle Temporal | 0.732 | 0.117 |  | 0.747 | 0.094 |  | 0.744 | 0.107 | 1.000 | 1.000 | 1.000 | 0.002 | 0.946 | 0.002 |
| Right Parahippocampal | 0.686 | 0.079 |  | 0.710 | 0.092 |  | 0.705 | 0.080 | 1.000 | 1.000 | 1.000 | 0.006 | 0.946 | 0.002 |
| Right Paracentral | 0.767 | 0.111 |  | 0.794 | 0.094 |  | 0.741 | 0.109 | 1.000 | 1.000 | 1.000 | 0.025 | 0.423 | 0.085 |
| Right Pars Opercularis | 0.792 | 0.112 |  | 0.756 | 0.128 |  | 0.746 | 0.120 | 1.000 | 1.000 | 1.000 | 0.014 | 0.907 | 0.011 |
| Right Pars Orbitalis | 0.860 | 0.134 |  | 0.829 | 0.117 |  | 0.888 | 0.078 | 1.000 | 1.000 | 1.000 | 0.004 | 0.907 | 0.018 |
| Right Pars Triangularis | 0.782 | 0.101 |  | 0.786 | 0.098 |  | 0.801 | 0.088 | 1.000 | 1.000 | 1.000 | 0.002 | 0.985 | 0 |
| Right Pericalcarine | 0.807 | 0.099 |  | 0.818 | 0.111 |  | 0.798 | 0.106 | 1.000 | 1.000 | 1.000 | 0.001 | 0.985 | 0 |
| Right Postcentral | 0.613 | 0.051 |  | 0.602 | 0.066 |  | 0.642 | 0.084 | 1.000 | 1.000 | 1.000 | 0.032 | 0.780 | 0.08 |
| Right Posterior Cingulate | 0.691 | 0.073 |  | 0.701 | 0.077 |  | 0.731 | 0.055 | 1.000 | 1.000 | 1.000 | 0.010 | 0.946 | 0.002 |
| Right Precentral | 0.567 | 0.045 |  | 0.562 | 0.043 |  | 0.565 | 0.067 | 1.000 | 1.000 | 1.000 | 0.003 | 0.946 | 0.005 |
| Right Precuneus | 0.545 | 0.044 |  | 0.520 | 0.054 |  | 0.539 | 0.062 | 1.000 | 1.000 | 1.000 | 0.009 | 0.907 | 0.021 |
| Right Rostral Anterior Cingulate | 0.709 | 0.092 |  | 0.715 | 0.084 |  | 0.706 | 0.081 | 1.000 | 1.000 | 1.000 | 0.001 | 0.907 | 0.011 |
| Right Rostral Middle Frontal | 0.714 | 0.096 |  | 0.726 | 0.132 |  | 0.705 | 0.119 | 1.000 | 1.000 | 1.000 | 0.000 | 0.946 | 0.003 |
| Right Superior Frontal | 0.451 | 0.040 |  | 0.456 | 0.050 |  | 0.459 | 0.043 | 1.000 | 1.000 | 1.000 | 0.002 | 0.946 | 0.005 |
| Right Superior Parietal | 0.505 | 0.059 |  | 0.485 | 0.059 |  | 0.500 | 0.101 | 1.000 | 1.000 | 1.000 | 0.010 | 0.907 | 0.022 |
| Right Supramarginal | 0.794 | 0.106 |  | 0.796 | 0.072 |  | 0.794 | 0.083 | 1.000 | 1.000 | 1.000 | 0.000 | 0.907 | 0.007 |
| Right Transverse Temporal | 0.963 | 0.064 |  | 0.940 | 0.083 |  | 0.953 | 0.063 | 1.000 | 1.000 | 1.000 | 0.003 | 0.946 | 0.001 |
| Right Insula | 0.642 | 0.079 |  | 0.616 | 0.071 |  | 0.655 | 0.077 | 1.000 | 1.000 | 1.000 | 0.012 | 0.946 | 0.003 |

* p<0.05, ** p<0.01, *** p<0.001, **** p<0.0001
