# Supplementary material for: Connectomic insights into the impact of 1p/19q co-deletion in dominant hemisphere insular glioma patients
Source: Front Neurosci. 2024 Jul 29;18:1283518. doi: 10.3389/fnins.2024.1283518 (PMC11317282; doi:10.3389/fnins.2024.1283518)
Supplement: Supplementary file 4 [file Table_4.docx]

**S-Table 4 The differences in betweenness centrality among healthy control, 1p/19q co-deletion, and 1p/19q non-co-deletion groups**

| **Regions** | **HC (n=20)** | |  | **1p/19q co-deletion patients (n=13)** | |  | **1p/19q non-co-deletion patients (n=19)** | | **FDR-corrected p-value (HC vs 1p/19q co-deletion patients)**, **controlling for age and sex** | **FDR-corrected p-value (HC vs 1p/19q non-co-deletion patients)**, **controlling for age and sex** | **FDR-corrected p-value (1p/19q co-deletion vs 1p/19q non-co-deletion patients)**, **controlling for age and sex** | **Cohen’s f^2^ (HC vs 1p/19q CD vs 1p/19q NCD patients)** | **FDR-corrected p-value (1p/19q co-deletion vs 1p/19q non-co-deletion patients)**, **controlling for age, sex, tumor volume, and grades** | **Cohen’s f^2^ (1p/19q CD vs 1p/19q NCD patients) , controlling for age, sex, tumor volume, and grades** |
| --- | --- | --- | --- | --- | --- | --- | --- | --- | --- | --- | --- | --- | --- | --- |
|  | **Mean** | **SD** |  | **Mean** | **SD** |  | **Mean** | **SD** |  |  |  |  |  |  |
| Left Caudal Anterior Cingulate | 10.621 | 7.396 |  | 10.857 | 22.644 |  | 5.136 | 5.540 | 1.000 | 1.000 | 1.000 | 0.068 | 0.694 | 0.113 |
| Left Caudal Middle Frontal | 3.534 | 4.079 |  | 7.502 | 7.815 |  | 6.158 | 6.159 | 1.000 | 1.000 | 1.000 | 0.070 | 0.976 | 0.011 |
| Left Cuneus | 29.029 | 25.011 |  | 26.759 | 25.376 |  | 30.718 | 31.384 | 1.000 | 1.000 | 1.000 | 0.008 | 0.976 | 0.003 |
| Left Entorhinal | 32.190 | 24.054 |  | 32.717 | 30.630 |  | 16.141 | 11.321 | 1.000 | 1.000 | 1.000 | 0.096 | 0.953 | 0.057 |
| Left Fusiform | 26.678 | 31.185 |  | 17.759 | 16.779 |  | 10.195 | 8.218 | 1.000 | 1.000 | 1.000 | 0.093 | 0.976 | 0.012 |
| Left Inferior Parietal | 15.823 | 15.023 |  | 24.217 | 18.979 |  | 19.594 | 15.285 | 1.000 | 1.000 | 1.000 | 0.028 | 0.976 | 0.009 |
| Left Inferior Temporal | 68.311 | 51.350 |  | 47.050 | 32.357 |  | 31.887 | 34.570 | 1.000 | 0.836 | 1.000 | 0.143 | 0.976 | 0.001 |
| Left Isthmus Cingulate | 33.308 | 26.363 |  | 26.372 | 12.179 |  | 36.493 | 25.778 | 1.000 | 1.000 | 1.000 | 0.011 | 0.877 | 0.080 |
| Left Lateral Occipital | 18.085 | 13.513 |  | 17.287 | 21.235 |  | 18.558 | 22.159 | 1.000 | 1.000 | 1.000 | 0.001 | 0.976 | 0.002 |
| Left Lateral Orbitofrontal | 52.717 | 24.947 |  | 84.265 | 35.505 |  | 78.744 | 38.464 | 1.000 | 0.843 | 1.000 | 0.164 | 0.976 | 0.001 |
| Left Lingual | 25.999 | 37.720 |  | 19.566 | 12.661 |  | 27.518 | 22.528 | 1.000 | 1.000 | 1.000 | 0.011 | 0.976 | 0.016 |
| Left Medial Orbitofrontal | 33.495 | 23.532 |  | 41.892 | 39.382 |  | 49.136 | 36.854 | 1.000 | 1.000 | 1.000 | 0.059 | 0.976 | 0.038 |
| Left Middle Temporal | 23.266 | 20.851 |  | 29.776 | 28.490 |  | 22.322 | 18.063 | 1.000 | 1.000 | 1.000 | 0.065 | 0.953 | 0.050 |
| Left Parahippocampal | 32.722 | 45.037 |  | 22.774 | 20.296 |  | 12.439 | 17.191 | 1.000 | 1.000 | 1.000 | 0.082 | 0.877 | 0.072 |
| Left Paracentral | 19.447 | 19.318 |  | 16.732 | 14.168 |  | 14.497 | 11.960 | 1.000 | 1.000 | 1.000 | 0.018 | 0.976 | 0.000 |
| Left Pars Opercularis | 11.645 | 9.677 |  | 22.098 | 20.987 |  | 17.377 | 17.792 | 1.000 | 1.000 | 1.000 | 0.070 | 0.976 | 0.028 |
| Left Pars Orbitalis | 2.610 | 6.118 |  | 5.835 | 9.184 |  | 11.683 | 14.334 | 1.000 | 0.836 | 1.000 | 0.145 | 0.976 | 0.018 |
| Left Pars Triangularis | 11.815 | 9.544 |  | 14.115 | 12.522 |  | 21.690 | 20.343 | 1.000 | 1.000 | 1.000 | 0.090 | 0.976 | 0.044 |
| Left Pericalcarine | 17.903 | 18.892 |  | 15.370 | 20.339 |  | 20.720 | 18.138 | 1.000 | 1.000 | 1.000 | 0.021 | 0.976 | 0.011 |
| Left Postcentral | 43.845 | 17.741 |  | 49.874 | 18.932 |  | 44.054 | 20.591 | 1.000 | 1.000 | 1.000 | 0.021 | 0.953 | 0.041 |
| Left Posterior Cingulate | 13.823 | 7.111 |  | 15.080 | 8.179 |  | 9.836 | 7.762 | 1.000 | 1.000 | 1.000 | 0.082 | 0.694 | 0.143 |
| Left Precentral | 69.067 | 41.163 |  | 74.481 | 53.610 |  | 75.348 | 36.823 | 1.000 | 1.000 | 1.000 | 0.007 | 0.976 | 0.009 |
| Left Precuneus | 107.607 | 40.090 |  | 105.851 | 43.092 |  | 109.578 | 35.157 | 1.000 | 1.000 | 1.000 | 0.000 | 0.976 | 0.004 |
| Left Rostral Anterior Cingulate | 17.476 | 20.416 |  | 15.482 | 9.415 |  | 18.972 | 13.315 | 1.000 | 1.000 | 1.000 | 0.006 | 0.976 | 0.024 |
| Left Rostral Middle Frontal | 24.322 | 20.658 |  | 21.691 | 18.649 |  | 35.229 | 26.259 | 1.000 | 1.000 | 0.843 | 0.116 | 0.694 | 0.147 |
| Left Superior Frontal | 208.851 | 74.202 |  | 120.490 | 57.312 |  | 103.792 | 66.187 | 0.183 | 0.001* | 1.000 | 0.429 | 0.976 | 0.010 |
| Left Superior Parietal | 80.453 | 50.982 |  | 104.406 | 49.465 |  | 92.015 | 59.217 | 1.000 | 1.000 | 1.000 | 0.054 | 0.976 | 0.003 |
| Left Superior Temporal | 123.932 | 58.140 |  | 103.674 | 54.659 |  | 69.348 | 46.354 | 1.000 | 0.183 | 0.905 | 0.185 | 0.626 | 0.210 |
| Left Supramarginal | 8.410 | 9.647 |  | 8.709 | 6.524 |  | 9.469 | 12.086 | 1.000 | 1.000 | 1.000 | 0.010 | 0.976 | 0.004 |
| Left Transverse Temporal | 0.710 | 0.984 |  | 2.360 | 4.319 |  | 5.922 | 7.271 | 1.000 | 0.361 | 1.000 | 0.201 | 0.976 | 0.022 |
| Left Insula | 49.000 | 49.039 |  | 24.235 | 30.090 |  | 24.920 | 27.521 | 1.000 | 1.000 | 1.000 | 0.093 | 0.976 | 0.007 |
| Right Superior Temporal | 85.882 | 52.947 |  | 73.434 | 41.664 |  | 82.153 | 34.362 | 1.000 | 1.000 | 1.000 | 0.004 | 0.976 | 0.032 |
| Right Caudal Anterior Cingulate | 14.722 | 16.842 |  | 10.501 | 8.104 |  | 14.456 | 10.466 | 1.000 | 1.000 | 1.000 | 0.009 | 0.976 | 0.012 |
| Right Caudal Middle Frontal | 5.941 | 6.480 |  | 3.842 | 6.883 |  | 5.113 | 8.324 | 1.000 | 1.000 | 1.000 | 0.011 | 0.976 | 0.000 |
| Right Cuneus | 22.949 | 21.775 |  | 37.613 | 36.265 |  | 30.299 | 22.384 | 1.000 | 1.000 | 1.000 | 0.044 | 0.976 | 0.029 |
| Right Entorhinal | 18.368 | 17.649 |  | 23.280 | 21.627 |  | 27.959 | 26.880 | 1.000 | 1.000 | 1.000 | 0.038 | 0.976 | 0.005 |
| Right Fusiform | 23.995 | 12.684 |  | 15.562 | 20.015 |  | 11.955 | 13.341 | 1.000 | 0.836 | 1.000 | 0.128 | 0.976 | 0.012 |
| Right Inferior Parietal | 25.435 | 22.482 |  | 29.740 | 20.677 |  | 26.770 | 30.423 | 1.000 | 1.000 | 1.000 | 0.020 | 0.976 | 0.014 |
| Right Inferior Temporal | 74.091 | 42.080 |  | 68.199 | 36.912 |  | 59.230 | 34.198 | 1.000 | 1.000 | 1.000 | 0.019 | 0.976 | 0.001 |
| Right Isthmus Cingulate | 28.374 | 40.168 |  | 39.975 | 29.640 |  | 27.120 | 12.641 | 1.000 | 1.000 | 1.000 | 0.045 | 0.976 | 0.044 |
| Right Lateral Occipital | 25.487 | 24.218 |  | 26.389 | 23.241 |  | 26.608 | 25.386 | 1.000 | 1.000 | 1.000 | 0.003 | 0.976 | 0.002 |
| Right Lateral Orbitofrontal | 81.136 | 47.803 |  | 95.132 | 52.989 |  | 68.092 | 40.774 | 1.000 | 1.000 | 1.000 | 0.008 | 0.976 | 0.008 |
| Right Lingual | 20.374 | 16.622 |  | 17.386 | 20.828 |  | 17.199 | 15.949 | 1.000 | 1.000 | 1.000 | 0.015 | 0.976 | 0.006 |
| Right Medial Orbitofrontal | 29.411 | 25.936 |  | 38.891 | 36.617 |  | 36.193 | 18.105 | 1.000 | 1.000 | 1.000 | 0.024 | 0.976 | 0.000 |
| Right Middle Temporal | 18.907 | 14.856 |  | 18.058 | 22.546 |  | 21.246 | 33.106 | 1.000 | 1.000 | 1.000 | 0.002 | 0.976 | 0.000 |
| Right Parahippocampal | 34.540 | 47.097 |  | 20.742 | 21.807 |  | 21.592 | 20.120 | 1.000 | 1.000 | 1.000 | 0.035 | 0.976 | 0.000 |
| Right Paracentral | 12.275 | 16.876 |  | 6.631 | 7.129 |  | 20.650 | 27.049 | 1.000 | 1.000 | 1.000 | 0.095 | 0.694 | 0.140 |
| Right Pars Opercularis | 11.629 | 17.915 |  | 12.174 | 9.582 |  | 15.291 | 14.925 | 1.000 | 1.000 | 1.000 | 0.014 | 0.877 | 0.076 |
| Right Pars Orbitalis | 5.680 | 9.755 |  | 7.258 | 9.967 |  | 3.072 | 4.562 | 1.000 | 1.000 | 1.000 | 0.015 | 0.976 | 0.027 |
| Right Pars Triangularis | 9.162 | 8.543 |  | 7.694 | 6.370 |  | 6.863 | 6.129 | 1.000 | 1.000 | 1.000 | 0.017 | 0.976 | 0.001 |
| Right Pericalcarine | 8.347 | 8.604 |  | 8.743 | 18.548 |  | 10.545 | 10.717 | 1.000 | 1.000 | 1.000 | 0.004 | 0.976 | 0.000 |
| Right Postcentral | 42.540 | 19.485 |  | 45.523 | 25.782 |  | 31.354 | 16.525 | 1.000 | 1.000 | 1.000 | 0.098 | 0.694 | 0.128 |
| Right Posterior Cingulate | 16.128 | 9.661 |  | 14.247 | 7.720 |  | 11.495 | 4.836 | 1.000 | 1.000 | 1.000 | 0.062 | 0.976 | 0.006 |
| Right Precentral | 61.805 | 25.087 |  | 64.481 | 27.766 |  | 69.479 | 30.428 | 1.000 | 1.000 | 1.000 | 0.007 | 0.976 | 0.004 |
| Right Precuneus | 88.837 | 48.536 |  | 115.807 | 65.362 |  | 109.704 | 68.425 | 1.000 | 1.000 | 1.000 | 0.029 | 0.976 | 0.004 |
| Right Rostral Anterior Cingulate | 20.760 | 22.229 |  | 13.538 | 8.048 |  | 18.052 | 15.003 | 1.000 | 1.000 | 1.000 | 0.037 | 0.877 | 0.091 |
| Right Rostral Middle Frontal | 17.155 | 13.594 |  | 15.336 | 13.919 |  | 21.438 | 24.709 | 1.000 | 1.000 | 1.000 | 0.018 | 0.976 | 0.022 |
| Right Superior Frontal | 231.995 | 60.699 |  | 228.310 | 112.719 |  | 249.331 | 112.015 | 1.000 | 1.000 | 1.000 | 0.004 | 0.976 | 0.000 |
| Right Superior Parietal | 148.051 | 71.190 |  | 174.559 | 96.011 |  | 197.512 | 114.692 | 1.000 | 1.000 | 1.000 | 0.047 | 0.976 | 0.000 |
| Right Supramarginal | 14.244 | 25.728 |  | 9.126 | 8.336 |  | 8.104 | 4.697 | 1.000 | 1.000 | 1.000 | 0.034 | 0.976 | 0.030 |
| Right Transverse Temporal | 1.020 | 3.764 |  | 1.423 | 2.520 |  | 0.450 | 0.699 | 1.000 | 1.000 | 1.000 | 0.034 | 0.877 | 0.078 |
| Right Insula | 40.464 | 43.613 |  | 44.518 | 23.063 |  | 29.503 | 16.141 | 1.000 | 1.000 | 1.000 | 0.039 | 0.976 | 0.024 |

* p<0.05, ** p<0.01, *** p<0.001, **** p<0.0001
