# Supplementary material for: Connectomic insights into the impact of 1p/19q co-deletion in dominant hemisphere insular glioma patients
Source: Front Neurosci. 2024 Jul 29;18:1283518. doi: 10.3389/fnins.2024.1283518 (PMC11317282; doi:10.3389/fnins.2024.1283518)
Supplement: Supplementary file 5 [file Table_5.docx]

**S-Table 5 Part One Statistical power comparison of global metrics - HC vs 1p/19q CD vs 1p/19q NCD**

| **Global metrics** | **Statistical power (%)** | |
| --- | --- | --- |
|  | **HC vs 1p/19q CD vs 1p/19q NCD** | **1p/19q CD vs 1p/19q NCD while for age, gender, tumor volume, and grade** |
| Density | 19.60 | 8.40 |
| Clustering coff (B) | 24.00 | 10.20 |
| Clustering coff (W) | 31.50 | 20.90 |
| Transitivity (B) | 54.80 | 14.20 |
| Transitivity (W) | 39.90 | 24.10 |
| Path Length (B) | 16.50 | 8.90 |
| Path Length (W) | 41.80 | 25.40 |
| Small-Worldness (B) | 30.60 | 13.90 |
| Small-Worldness (W) | 25.20 | 25.30 |
| Global efficiency (B) | 18.50 | 8.70 |
| Global efficiency (W) | 33.90 | 33.70 |
| Diameter of graph (B) | 13.60 | 11.50 |
| Diameter of graph (W) | 18.30 | 5.70 |
| Radius of graph (B) | 15.70 | 7.70 |
| Radius of graph (W) | 16.60 | 5.40 |
| Assortativity coff (B) | 33.20 | 14.10 |
| Assortativity coff (W) | 47.20 | 6.80 |
| Rich club K5 (B) | 17.30 | 7.60 |
| Rich club K10 (B) | 8.90 | 7.20 |
| Rich club K15 (B) | 18.70 | 14.50 |
| Rich club K20 (B) | 8.20 | 6.20 |
| Rich club K25 (B) | 13.60 | 9.40 |
| Rich club K5 (W) | 30.30 | 20.40 |
| Rich club K10 (W) | 38.00 | 11.60 |
| Rich club K15 (W) | 10.40 | 5.40 |
| Rich club K20 (W) | 16.10 | 9.20 |
| Rich club K25 (W) | 21.40 | 6.70 |

HC = Healthy Controls, 1p/19q CD = 1p/19q Co-deletion, 1p/19q NCD = 1p/19q Non-co-deletion

**S-Table 5 Part Two Statistical power comparison of local metrics - HC vs 1p/19q CD vs 1p/19q NCD**

| **Local metrics** | **Statistical power (%)** | | | | |
| --- | --- | --- | --- | --- | --- |
|  | **Clustering coefficient** | |  | **Betweenness centrality** | |
|  | **HC vs 1p/19q CD vs 1p/19q NCD** | **1p/19q CD vs 1p/19q NCD while for age, gender, tumor volume, and grade** |  | **HC vs 1p/19q CD vs 1p/19q NCD** | **1p/19q CD vs 1p/19q NCD while for age, gender, tumor volume, and grade** |
| Left Caudal Anterior Cingulate | 67.50 | 15.20 |  | 29.60 | 22.00 |
| Left Caudal Middle Frontal | 14.30 | 7.90 |  | 30.50 | 6.30 |
| Left Cuneus | 5.50 | 5.20 |  | 7.50 | 5.30 |
| Left Entorhinal | 16.30 | 7.50 |  | 40.70 | 12.60 |
| Left Fusiform | 13.90 | 5.00 |  | 39.60 | 6.40 |
| Left Inferior Parietal | 7.70 | 5.30 |  | 14.20 | 6.00 |
| Left Inferior Temporal | 29.40 | 7.10 |  | 57.90 | 5.10 |
| Left Isthmus Cingulate | 6.00 | 5.20 |  | 8.30 | 16.40 |
| Left Lateral Occipital | 6.60 | 5.40 |  | 5.20 | 5.20 |
| Left Lateral Orbitofrontal | 10.80 | 5.70 |  | 64.50 | 5.10 |
| Left Lingual | 8.70 | 5.10 |  | 8.20 | 6.90 |
| Left Medial Orbitofrontal | 8.70 | 7.40 |  | 26.10 | 9.90 |
| Left Middle Temporal | 15.80 | 7.30 |  | 28.60 | 11.60 |
| Left Parahippocampal | 54.90 | 27.00 |  | 35.20 | 15.00 |
| Left Paracentral | 7.20 | 5.20 |  | 10.60 | 5.00 |
| Left Pars Opercularis | 6.20 | 7.60 |  | 30.40 | 8.40 |
| Left Pars Orbitalis | 25.90 | 7.10 |  | 58.80 | 7.20 |
| Left Pars Triangularis | 7.90 | 6.60 |  | 38.30 | 10.60 |
| Left Pericalcarine | 11.20 | 7.90 |  | 11.60 | 6.30 |
| Left Postcentral | 10.90 | 13.80 |  | 11.70 | 10.30 |
| Left Posterior Cingulate | 39.40 | 21.40 |  | 35.30 | 27.40 |
| Left Precentral | 7.00 | 5.00 |  | 7.10 | 6.00 |
| Left Precuneus | 5.90 | 6.00 |  | 5.10 | 5.50 |
| Left Rostral Anterior Cingulate | 5.20 | 5.40 |  | 6.70 | 8.00 |
| Left Rostral Middle Frontal | 13.00 | 11.20 |  | 48.40 | 28.10 |
| Left Superior Frontal | 76.80 | 12.30 |  | 97.80 | 6.20 |
| Left Superior Parietal | 11.20 | 5.90 |  | 24.00 | 5.30 |
| Left Superior Temporal | 63.50 | 29.90 |  | 70.30 | 39.70 |
| Left Supramarginal | 6.00 | 6.60 |  | 8.00 | 5.50 |
| Left Transverse Temporal | 29.30 | 6.60 |  | 74.40 | 7.60 |
| Left Insula | 70.10 | 7.20 |  | 39.60 | 5.80 |
| Right Superior Temporal | 8.70 | 8.50 |  | 6.10 | 9.00 |
| Right Caudal Anterior Cingulate | 5.70 | 5.30 |  | 7.70 | 6.40 |
| Right Caudal Middle Frontal | 6.10 | 5.00 |  | 8.20 | 5.00 |
| Right Cuneus | 11.60 | 6.20 |  | 20.00 | 8.60 |
| Right Entorhinal | 5.30 | 5.00 |  | 17.70 | 5.60 |
| Right Fusiform | 40.50 | 5.10 |  | 52.70 | 6.40 |
| Right Inferior Parietal | 14.80 | 11.90 |  | 11.30 | 6.60 |
| Right Inferior Temporal | 5.50 | 5.00 |  | 11.10 | 5.20 |
| Right Isthmus Cingulate | 18.20 | 6.60 |  | 20.40 | 10.70 |
| Right Lateral Occipital | 9.50 | 8.40 |  | 5.90 | 5.20 |
| Right Lateral Orbitofrontal | 5.10 | 5.10 |  | 7.30 | 5.90 |
| Right Lingual | 8.40 | 5.00 |  | 9.70 | 5.70 |
| Right Medial Orbitofrontal | 12.90 | 6.60 |  | 12.70 | 5.00 |
| Right Middle Temporal | 5.50 | 5.30 |  | 5.50 | 5.10 |
| Right Parahippocampal | 6.80 | 5.20 |  | 16.70 | 5.00 |
| Right Paracentral | 12.90 | 17.10 |  | 40.60 | 26.90 |
| Right Pars Opercularis | 9.10 | 6.30 |  | 9.30 | 15.60 |
| Right Pars Orbitalis | 6.00 | 7.20 |  | 9.60 | 8.40 |
| Right Pars Triangularis | 5.60 | 5.00 |  | 10.20 | 5.10 |
| Right Pericalcarine | 32.046 | 5.00 |  | 6.20 | 5.00 |
| Right Postcentral | 42.710 | 16.20 |  | 41.40 | 24.60 |
| Right Posterior Cingulate | 83.741 | 5.20 |  | 27.30 | 5.60 |
| Right Precentral | 35.035 | 5.60 |  | 7.20 | 5.50 |
| Right Precuneus | 21.389 | 7.60 |  | 14.50 | 5.50 |
| Right Rostral Anterior Cingulate | 6.616 | 6.30 |  | 17.30 | 18.10 |
| Right Rostral Middle Frontal | 16.136 | 5.30 |  | 10.50 | 7.60 |
| Right Superior Frontal | 9.344 | 5.60 |  | 6.20 | 5.00 |
| Right Superior Parietal | 20.471 | 7.60 |  | 21.50 | 5.00 |
| Right Supramarginal | 6.313 | 5.80 |  | 16.50 | 8.70 |
| Right Transverse Temporal | 13.417 | 5.10 |  | 16.20 | 16.10 |
| Right Insula | 22.982 | 5.40 |  | 18.00 | 7.90 |

HC = Healthy Controls, 1p/19q CD = 1p/19q Co-deletion, 1p/19q NCD = 1p/19q Non-co-deletion
